# Construction and validation of a T cell proliferation regulator-related signature for predicting prognosis and immunotherapy response in lung adenocarcinoma

**DOI:** 10.3389/fimmu.2023.1171145

**Published:** 2023-04-04

**Authors:** Wuguang Chang, Hongmu Li, Yixin Cheng, Huanhuan He, Wei Ou, Si-Yu Wang

**Affiliations:** ^1^ Department of Thoracic Surgery, Sun Yat-sen University Cancer Center, State Key Laboratory of Oncology in South China, Collaborative Innovation Center for Cancer Medicine, Guangzhou, China; ^2^ Department of Radiation Oncology, Sun Yat-sen University Cancer Center, State Key Laboratory of Oncology in South China, Collaborative Innovation Center of Cancer Medicine, Guangzhou, China; ^3^ Department of Pathology, State Key Laboratory of Oncology in South China, Collaborative Innovation Center for Cancer Medicine, Guangzhou, China

**Keywords:** T cell proliferation regulator, lung adenocarcinoma, biomarker, tumor microenvironment, ADA, immunotherapy

## Abstract

**Background:**

As the main executor of immunotherapy, T cells significantly affect the efficacy of immunotherapy. However, the contribution of the T cell proliferation regulator to the prognosis of lung adenocarcinoma (LUAD) and immunotherapy is still unclear.

**Methods:**

Based on T cell proliferation regulators, LUAD samples from The Cancer Genome Atlas (TCGA) were divided into two different clusters by consensus clustering. Subsequently, the T cell proliferation regulator (TPR) signature was constructed according to the prognostic T cell proliferation regulators. Combined with clinical information, a nomogram for clinical practice was constructed. The predictive ability of the signature was verified by the additional Gene Expression Omnibus (GEO) dataset. We also analyzed the differences of tumor microenvironment (TME) in different subgroups and predicted the response to immunotherapy according to the TIDE algorithm. Finally, we further explored the role of *ADA* (Adenosine deaminase) in the lung adenocarcinoma cell lines through the knockdown of *ADA*.

**Results:**

According to the consensus clustering, there were differences in survival and tumor microenvironment between two different molecular subtypes. T cell proliferation regulator-related signature could accurately predict the prognosis of LUAD. The low-risk group had a higher level of immune infiltration and more abundant immune-related pathways, and its response to immunotherapy was significantly better than the high-risk group (Chi-square test, p<0.0001). The knockdown of *ADA* inhibited proliferation, migration, and invasion in lung adenocarcinoma cell lines.

**Conclusion:**

T cell proliferation regulators were closely related to the prognosis and tumor microenvironment of LUAD patients. And the signature could well predict the prognosis of LUAD patients and their response to immunotherapy. *ADA* may become a new target for the treatment of LUAD.

## 1 Introduction

Lung cancer is one of the malignant tumors with the highest incidence rate and mortality rate, and about 1.6 million people die of lung cancer every year in the world (1). With the increase in people’s understanding of the molecular level, the treatment of lung cancer has undergone profound changes. Compared with other pathological types of non-small cell lung cancer (NSCLC), LUAD has many available targets (2). The rapid development of targeted therapy and immunotherapy has brought good news to LUAD patients (3). However, due to the heterogeneity of tumor, whether targeted therapy or immunotherapy, drug resistance will inevitably occur eventually (4, 5). Therefore, there is an urgent need to explore new biomarkers to evaluate the prognosis of LUAD patients and thus prolong their survival.

T cells are the main effectors of cellular immunity, which mainly include two subgroups: CD4 expressing T helper (Th) cell and CD8 expressing cytotoxic T lymphocyte (CTL) (6). Th cells regulate immune response by secreting various cytokines (7), while CTL directly kills tumor cells as effector cells (8). In addition, regulatory T cells (Treg) play an immunosuppressive role (9). The purpose of immune checkpoint inhibitors (ICIs) is to reactivate T cells in the autoimmune system and kill tumor cells. However, most patients did not respond to immunotherapy (10). Low T cell infiltration was called cold tumors, which may lead to the failure of immunotherapy. By increasing the infiltration of T cells, cold tumors could be transformed into hot tumors and reverse the low reactivity to immunotherapy (11). Exploring the role of T cell proliferation regulators in cancer may have an important impact on anti-tumor immunity.

In this study, we used T cell proliferation regulators to build the TPR signature, which could well evaluate the prognosis of LUAD. In addition, the analysis of the tumor microenvironment showed that there were significant differences in immune cells and function between the two subgroups. More importantly, the signature can guide the immunotherapy of LUAD patients and provide new insights for individualized treatment. Cytological experiments confirmed the role of *ADA* in LUAD.

## 2 Material and methods

### 2.1 Data collection and processing

The transcriptome data of LUAD comes from TCGA, and both Count and TPM were collected. The count format was used for difference analysis, and the TPM format was converted to log_2_(TPM+1) for subsequent analysis. TCGA-LUAD cohort was used as training sets. To reduce the impact of non-tumor factors, we excluded the samples with missing survival data and overall survival (OS) of less than 30 days. Finally, we collected 485 LUAD and 59 normal samples. 398 LUAD patients from GSE72094 were used as validation sets. 35 T cell proliferation regulators were from previous studies (12).

### 2.2 Consensus cluster analysis

Based on the expression of T cell proliferation regulators, the TCGA cohort was classified using the consensus clustering algorithm, which was performed through ‘ConensusClusterPlus’ package (13). Using agglomerative pam clustering with a 1-pearson correlation distance and resampling 80% of the samples for 1000 repetitions to ensure the stability of the classification. The optimal number of clusters was determined using the empirical cumulative distribution function plot.

### 2.3 Function and pathway enrichment analysis

Based on the ‘limma’ package (14), the differential genes between different clusters were identified (p-value < 0.05 and log2 | fold change | > 1), and then the GO/KEGG enrichment analysis of these genes was carried out through the ‘clusterProfiler’ package (15).

### 2.4 Construction and validation of the T cell proliferation regulator signature

Differentially expressed T cell proliferation regulators (Fold Change=1.5, false discovery rate, FDR < 0.05) were identified by differential analysis. Then, the T cell proliferation regulators related to OS were further screened by univariate cox regression analysis, and they were included in the LASSO regression analysis in the next step. LASSO regression is a generalized linear model, which can reduce the overfitting of variables. When lambda takes the minimum value, the best genes and their coefficient are obtained. Risk score = Gene_A_*exp_A_+Gene_B_*exp_B_+ … Gene_n_*exp_n_. Then the patients were divided into the high- and low-risk group according to the median risk score. Kaplan-Meier (KM) survival analysis was used to test the survival difference between the two subgroups. In addition, we utilized ROC curves to compare the effectiveness of our model with previous studies (16–19).

### 2.5 Establishment and evaluation of the nomogram

To evaluate whether the TPR signature was affected by other clinical factors, we conducted the cox regression analysis based on the signature and combined them to build a nomogram through ‘regplot’ package. The nomogram’s prediction ability was evaluated by the ROC curve and calibration curve.

### 2.6 Gene set enrichment analysis

To further understand the differences in biological functions between different subtypes, GSEA (version 4.2.3) software was used for GO/KEGG enrichment analysis (20). The threshold value was set at p < 0.05 and FDR < 0.25.

### 2.7 Assessment of tumor microenvironment

Single sample gene set enrichment analysis (ssGSEA) is an extension of GSEA method, which calculates the enrichment fraction of each sample and gene set pair (21). We used the dataset related to immune cell markers to carry out ssGSEA through ‘GSVA’ R package. The ESTIMATE algorithm is a method that uses the expression of gene to infer the proportion of the intermediate and immune cells in tumor samples (22). We predict the immune score and matrix score of LUAD through the ‘estimate’ package. TISCH is an online single-cell database focused on tumor microenvironment (23). GSE131970 dataset contains 44 LUAD samples, totaling 203298 cells. According to the information provided by this database, the uniform manifest approximation and projection (UMAP) were utilized to reduce the dimension further and visualize the clustering results. Based on the TISCH database, we analyzed the expression of the key genes constituting the signature in the GSE131907 dataset. The scMetabolism is a calculation method for quantifying single-cell metabolism. We downloaded single-cell data containing three LUAD samples from GSE117570 and calculated T cell-related metabolic pathways at the single-cell level through the scMetabolism algorithm (24).

### 2.8 Prediction of immunotherapy

On the ground of the expression of transcriptome data, we used Tumor Immune Dysfunction and Exclusion (TIDE) algorithm to evaluate the response of LUAD patients to immunotherapy (25). The higher TIDE score means that patients are more prone to immune escape during immunotherapy.

### 2.9 Chemotherapy sensitivity analysis

The half-maximal inhibitory concentration (IC50) of common chemotherapeutic drugs for LUAD was calculated by ‘pRRophetic’ package, which was derived from the expression matrix and drug response information in the Cancer Genome Project (CGP) plan, including 138 anticancer drugs against 727 cell lines (26).

### 2.10 Cell culture and small-interfering RNA transfection

Xinyuan Biotech Co. Ltd. (Shanghai, China) provided the lung adenocarcinoma cell lines H1299 and PC9 with authentication using short tandem repeat profiling. DMEM (Gibco, Grand Island, NY, USA) containing 10% fetal bovine serum (Sigma, USA) and 5% CO_2_ was used to culture H1299 and PC9 cells. The sequences of siRNA targeting ADA were cloned into H1299 and PC9 cells. Using Lipofectamine 3000 (Invitrogen, USA), the siRNA transfection process was conducted as instructed by the manufacturer.

### 2.11 Western blotting

RIPA buffer (Promoter, Wuhan, China) with PMSF and protease inhibitor cocktail (MCE, USA) were used for dissociating the protein. BCA kit (Servicebio, Wuhan, China) was used to measure protein concentration in the supernatant after removing the precipitate. Electrophoresis was used to transfer the protein samples onto PVDF membranes after boiling the protein. At 4°C overnight, the membranes were incubated with primary antibodies. The membranes were washed three times with TBST and then incubated with secondary antibodies for one hour. The antibodies used in this study were as follows: anti-ADA (Proteintech, 17479-1-AP), anti-GAPDH (Proteintech, 60004-1-Ig).

### 2.12 Cell counting kit-8 assay

Cell Counting Kit-8 (CCK-8) was used to measure cell proliferation (JingXin Biological Technology, Guangzhou, China). Cell suspensions with a density of 5 x 105 cells/ml of H1299 and PC9 cells were prepared. 96-well plates (1 × 10^3^ cells/well) were filled with cell suspension (0.2 ml) and cultured in a 5% CO_2_ incubator at 37°C. The culture was repeated for 1, 2, 3, 4, and 5 days. Before measuring the absorbance, add 10 μl of CCK-8 solution to each well. With a microplate reader, the absorbance at 450 nm was measured after incubation for 2 hours.

### 2.13 Migration and invasion testing

Transwell plate (Corning, NY, USA) with 8-um pores was used to perform migration assays *in vitro*. Cells (5×10^4^) in serum-free DMEM medium (200ul) were placed on the upper layer of the chamber, and the medium containing 10% FBS was added to the lower chamber. After 36 hours of culture, cells in the upper chamber were removed, and cells in the lower chamber were stained and counted. Matrigel coating chambers (BD Biosciences) are used for invasion assay, cells (1.5×10^5^) in serum-free DMEM medium (200ul) were placed on the upper layer of the chamber, and the medium containing 10% FBS was added to the lower chamber. After 36 hours of culture, cells in the upper chamber were removed, and cells in the lower chamber were stained and counted. Four visual fields were taken in each plate, and the cells were counted with Image J. Each experiment was repeated three times.

### 2.14 Statistical analysis

All data analysis and graphs were completed by R software (version: 4.13). The chi-square test was used for categorical variables. The continuous variable was used by Wilcoxon test. Kaplan-Meier analysis and Log-rank test were used for survival assessment. Risk factors for LUAD were established by cox proportional hazards regression analysis. Only p < 0.05 was considered statistically significant.

## 3 Results

### 3.1 Characteristics of T cell proliferation regulators

**Figure 1:**
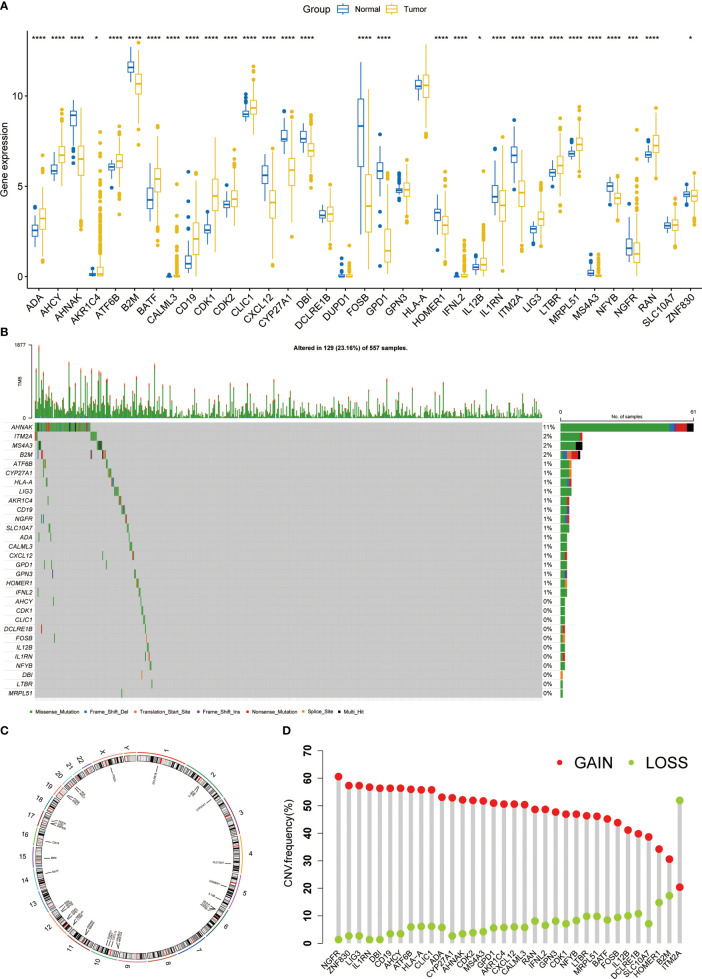
The expression and genomic features of T cell proliferation regulators in LUAD. **(A)** The differential expression of T cell proliferation regulators between tumor and normal samples. **(B)** Mutation landscape of T cell proliferation regulators in TCGA. **(C)** Chromosome position and alteration of T cell proliferation regulators. **(D)** The CNV mutation frequency of 35 T cell proliferation regulators. *p < 0.05, ***p < 0.001, ****p < 0.0001.

First, we explored the expression of T cell proliferation regulators in LUAD and normal tissues. Most of them showed clear differences (**Figure 1A**). A total of 23.16% of LUAD had mutations of T cell proliferation regulators, of which *AHNAK* had the highest mutation rate, reaching 11% (**Figure 1B**). The position of 35 T cell proliferation regulators on chromosomes was shown in **Figure 1C**. Through the analysis of CNV in TCGA database, we found that except *ITM2A*, other genes had a higher proportion of CNV gain (**Figure 1D**).

### 3.2 Consensus clustering and GO/KEGG enrichment analysis

**Figure 2:**
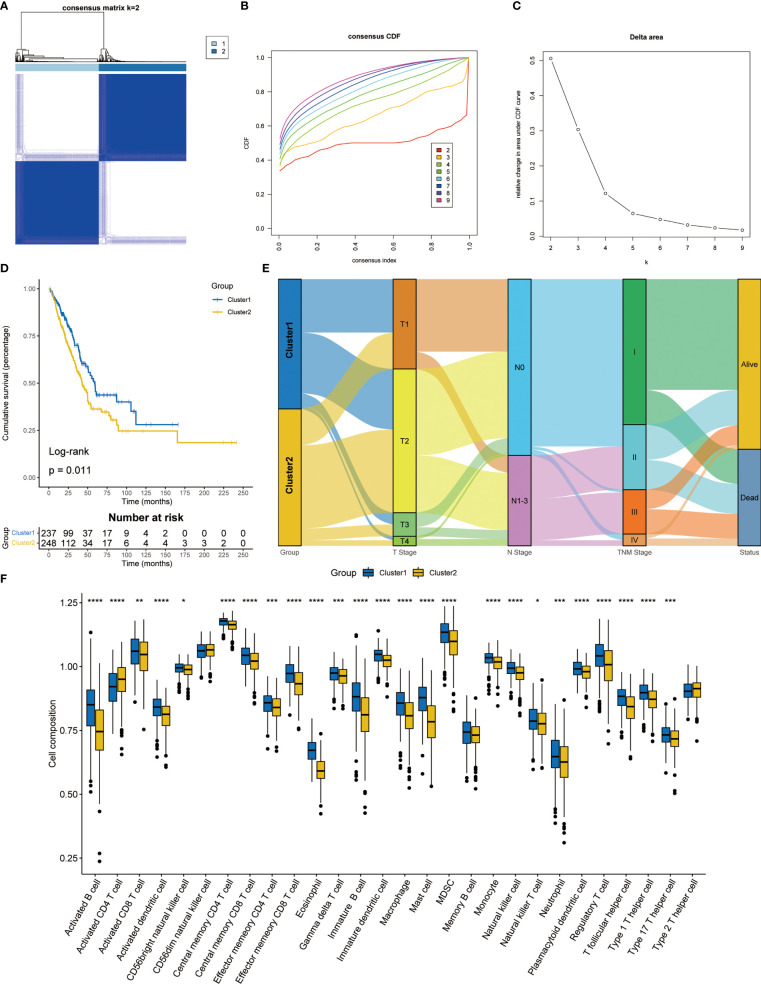
Consensus clustering analysis based on T cell proliferation regulators. **(A)** Consensus clustering matrix at k = 2. **(B)** The CDF curves for clusters from k = 2 to 9. **(C)** The relative change in the area under the CDF curve from k = 2 to 9. **(D)** Kaplan-Meier survival analysis between two clusters. **(E)** Sankey diagram showed the relationship between TNM stage and two clusters. **(F)** The proportion of 28 kinds of immune cells in two clusters. *p < 0.05, **p < 0.01, ***p < 0.001, ****p < 0.0001.

Consensus clustering was used to classify LUAD patients based on the expression levels of T cell proliferation regulators. According to the consensus matrix (**Figure 2A**), consensus CDF curves (**Figure 2B**), and relative change in the area under the CDF curves, the optimal division (K = 2) represented the ideal number of clusters (**Figure 2C**), indicating that patients could be well segregated into the two molecular subtypes. The expression of T cell proliferation regulators showed significant differences between the two clusters (**Figure S1**). More importantly, a significant difference of survival was found between the two subtypes (p = 0.011, **Figure 2D**). The relationship between the TNM stage and clusters was shown by Sankey diagram (**Figure 2E**). To explore the differences of potential pathways between different clusters, we analyzed the differentially expressed genes (DEGs) between the two clusters (p < 0.05, **Table S1**). *FGB*, *MAGEA3*, and *MAGEA6* were the most significantly upregulated. The *SFTPC*, *SCGB1A1*, and *ADH1B* with the most significant downregulate. Then, GO and KEGG enrichment analysis of these genes were carried out. GO enrichment analysis showed that in Cluster2, the up-regulated DEGs were closely related to the cell proliferation pathway (**Figure S2A**), and KEGG enrichment analysis also obtained similar results (**Figure S2B**). In Cluster1, GO enrichment analysis found that a large number of immune-related pathways were activated (**Figure S2C**). KEGG enrichment analysis showed that the up-regulated DEGs were mainly concentrated in the complement, coagulation pathway, and cell adhesion-related pathways (**Figure S2D**). These results showed that the activation of the tumor proliferation process was the main feature of Cluster2, and the activation of immune system was the main feature of Cluster1. Then we used ssGSEA to analyze the differences between the two clusters of 28 kinds of immune cells and found that almost all immune cells increased significantly in Cluster1 (**Figure 2F**), which just confirmed the results of enrichment analysis.

### 3.3 Development and validation of the T cell proliferation regulator signature

**Figure 3:**
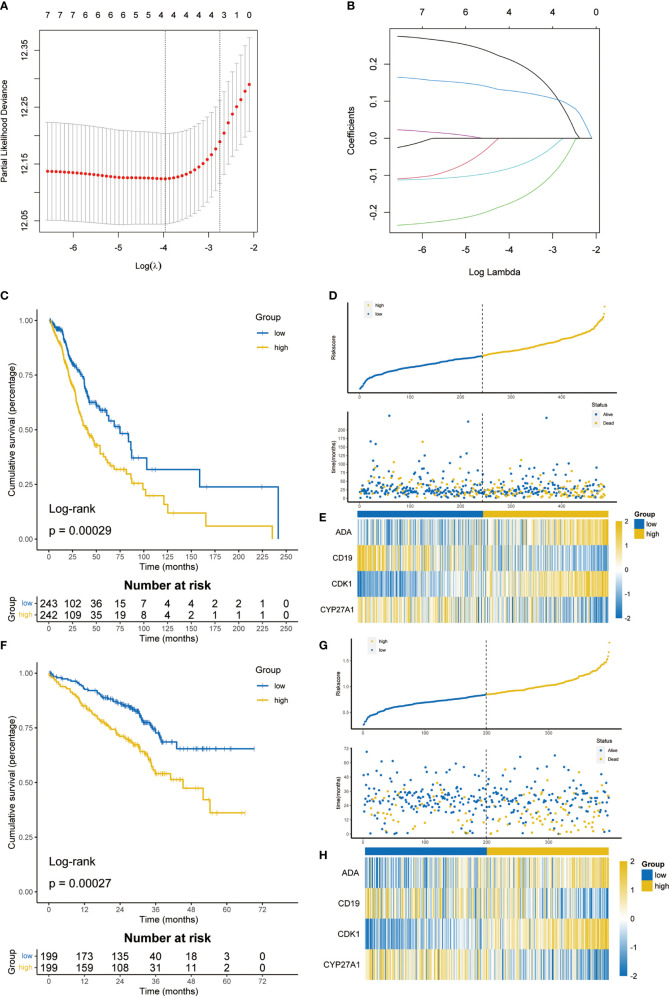
Construction and validation of T cell proliferation signature. **(A)** 10-fold cross-validation in the LASSO model. **(B)** Coefficient distribution of key T cell proliferation regulators. **(C)** The K-M survival analysis showed the difference of prognosis in TCGA cohort. **(D)** Distribution of risk score and survival status in TCGA cohort. **(E)** Heatmap showing 4 T cell proliferation regulators in TCGA cohort. **(F)** The K-M survival analysis showed the difference of prognosis in GSE72094. **(G)** Distribution of risk score and survival status in GSE72094. **(H)** Heatmap showing 4 T cell proliferation regulators in GSE72094.

Among 35 T cell proliferation regulators, 20 were differentially expressed. Subsequently, the genes related to OS were screened out through univariate cox regression analysis (**Figure S3**) and included in the LASSO Cox regression model (**Figures 3A, B**). Finally, four key genes and their coefficients were obtained. The risk score of each LUAD patient = ADA*0.21074646-CD19*0.17404017+CDK1*0.12803755-CYP27A2*0.08014371. On the basis of the median risk score, LUAD patients were divided into high-risk and low-risk groups. The prognosis of the high-risk group was significantly better than that of the low-risk group (p=0.00029, **Figure 3C**), and there were significantly more deaths in the high-risk group (**Figure 3D**). The heatmap showed the expression difference of four key T cell proliferation regulators between the two subgroups (**Figure 3E**). The prediction ability of signature had been further verified in the GSE72094 dataset, and the low-risk group had better OS (p = 0.00027, **Figure 3F**). The distribution of risk score and the expression of four key genes were almost consistent with the training set (**Figures 3G, H**). Furthermore, we compared the TPR signature with others’ signatures in the past, and the results showed that our signature has quite good prediction ability (**Figure S4**).

### 3.4 Establishment of the nomogram

**Figure 4:**
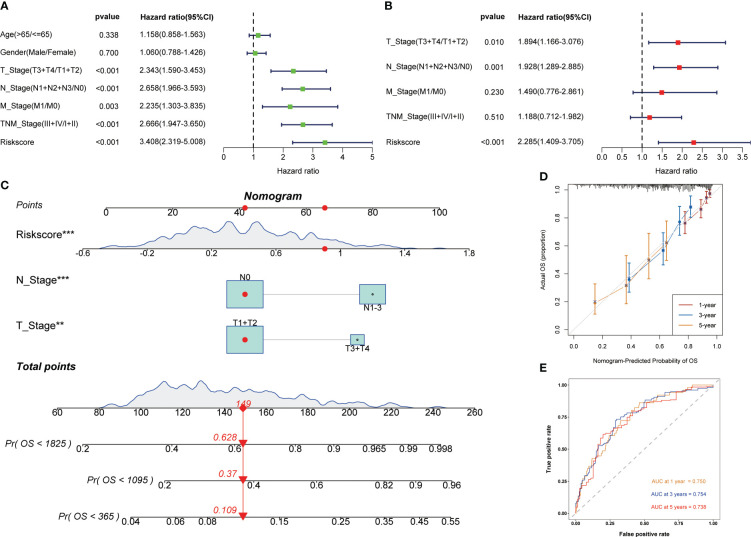
Development of the nomogram to predict the prognosis. Univariate **(A)** and multivariate **(B)** Cox regression analysis. **(C)** Nomogram for the prediction of 1-, 3- and 5-year survival probability. **(D)** Calibration curves for evaluating the accuracy. **(E)** ROC curves of the nomogram. **p < 0.01, ***p < 0.001.

Univariate and multivariate cox regression analysis showed that signature was a reliable prognostic marker (**Figures 4A, B**). Then, based on clinicopathological features and risk score, the nomogram was constructed to predict the 1-, 3-, and 5-year survival rate of LUAD patients (**Figure 4C**). The correction curve showed that the predicted results were highly consistent with the actual results (**Figure 4D**). The area under curves (AUC) of the nomogram in 1-, 3-, 5-year were 0.750, 0.754, and 0.738 respectively (**Figure 4E**). The above results showed that the nomogram had excellent prediction ability.

### 3.5 Gene set enrichment analysis

**Figure 5:**
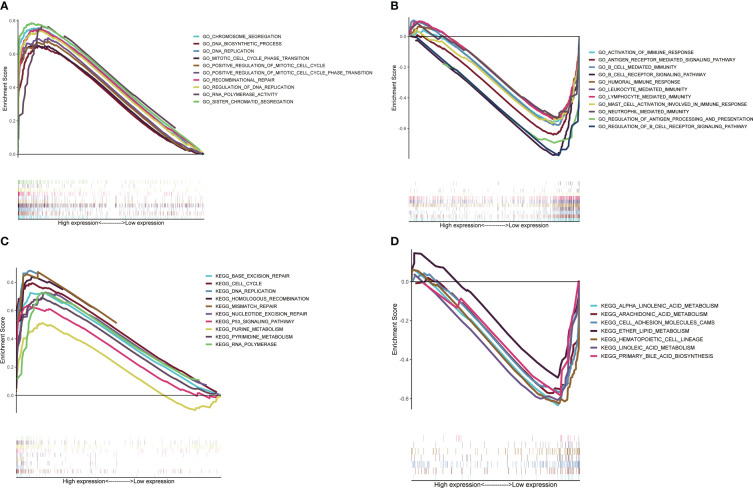
Potential pathways analysis based on GSEA. Analysis of GO enrichment in high-risk group **(A)** and low-risk group **(B)**. Analysis of KEGG enrichment in high-risk group **(C)** and low-risk group **(D)**.

To explore the difference in potential pathways between different subgroups of signature, we used GSEA to conduct GO/KEGG enrichment analysis. The GO enrichment analysis results showed that the high-risk groups were mainly enriched in DNA replication and cell proliferation pathways (**Figure 5A**), while the KEGG results showed similar results (**Figure 5C**). As mentioned above, T cell proliferation regulators and immune-related pathways were closely related, and GO enrichment analysis of the low-risk group confirmed this result (**Figure 5B**). The KEGG results showed that the low-risk group was closely related to the metabolism of multiple nutrients (**Figure 5D**).

### 3.6 Difference in the tumor microenvironment and prediction of immunotherapy response

**Figure 6:**
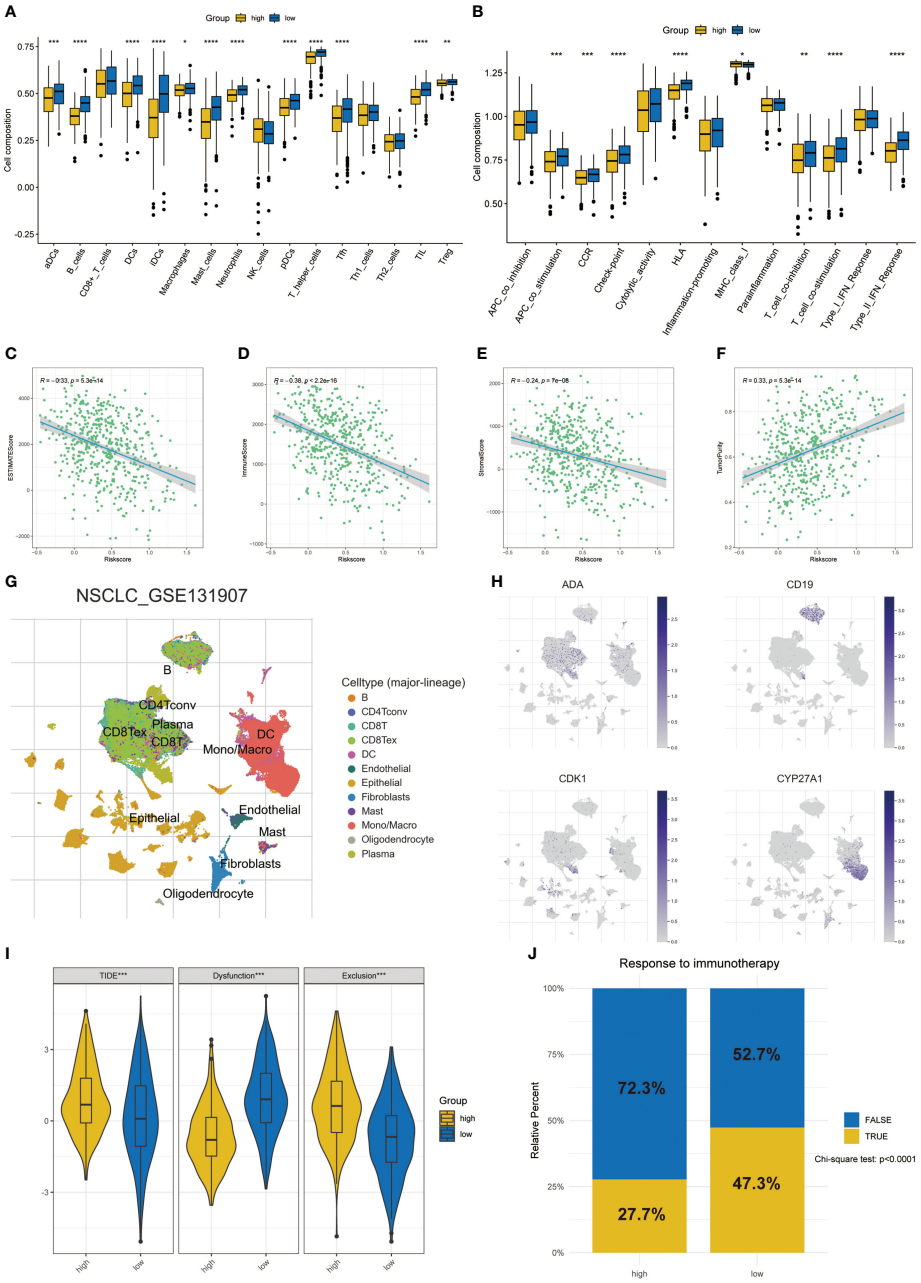
Differences of TME in two risk groups. **(A)** The differences in the proportions of 16 immune cells. **(B)** The differences of 13 immune-related pathways. Correlation between risk score and ESTIMATE score **(C)**, immune score **(D)**, stromal score **(E)**, and tumor purity **(F)** in two groups. **(G)** The annotation of celltypes in GSE131907. **(H)** The expression of key T cell proliferation regulators in GSE131907. **(I)** The differences of TIDE score. **(J)** Response to immunotherapy based on the TIDE algorithm. *p < 0.05, **p < 0.01, ***p < 0.001, ****p < 0.0001.

The state of tumor microenvironment significantly affects the development of tumor and the efficacy of immunotherapy. We used ssGSEA to analyze the differences between 16 immune cells and 13 immune-related pathways. In the low-risk group, many kinds of immune cells and pathways were enriched, which were in the state of immune activation (**Figures 6A, B**). In addition, the ESTIMATE algorithm showed that the risk score was significantly negatively correlated with the ESTIMATE score, immune score, and normal score, while it was significantly positively correlated with the tumor purity (**Figures 6C–F**). Next, we used the GSE131907 dataset in the TISCH database to analyze the differences of the four key T cell proliferation regulators in TME at the single cell level. The distribution of various cell types was shown in **Figure 6G**. ADA was mainly expressed in CD8T, CD8Tex, and plasma; CD19 was mainly expressed in B cell; CDK1 was mainly expressed in plasma and epinephrine cell; CYP27A1 was mainly distributed in Mono/Macro, DC, and Fibroblasts (**Figure 6H**). In addition, we used scMetabolism to explore T cell-related metabolic pathways and the results indicated that T cell were closely related to the starch and sucrose metabolism at the single-cell level (**Figure S5**). Subsequently, we calculated the TIDE score of each LUAD patient using the TIDE algorithm. The high-risk group had a higher TIDE score and exclusion, which indicated that the high-risk group was more likely to have immune escape (**Figure 6I**). In the prediction of the efficacy of immunotherapy, we found that a higher proportion of people in the low-risk group responded to immunotherapy (47.3% vs 27.7%, χ^2^ test: p < 0.0001, **Figure 6J**). These results meant that the low-risk group was in the state of immune activation, and thus had a better response to immunotherapy.

### 3.7 Chemotherapy sensitivity analysis

**Figure 7:**
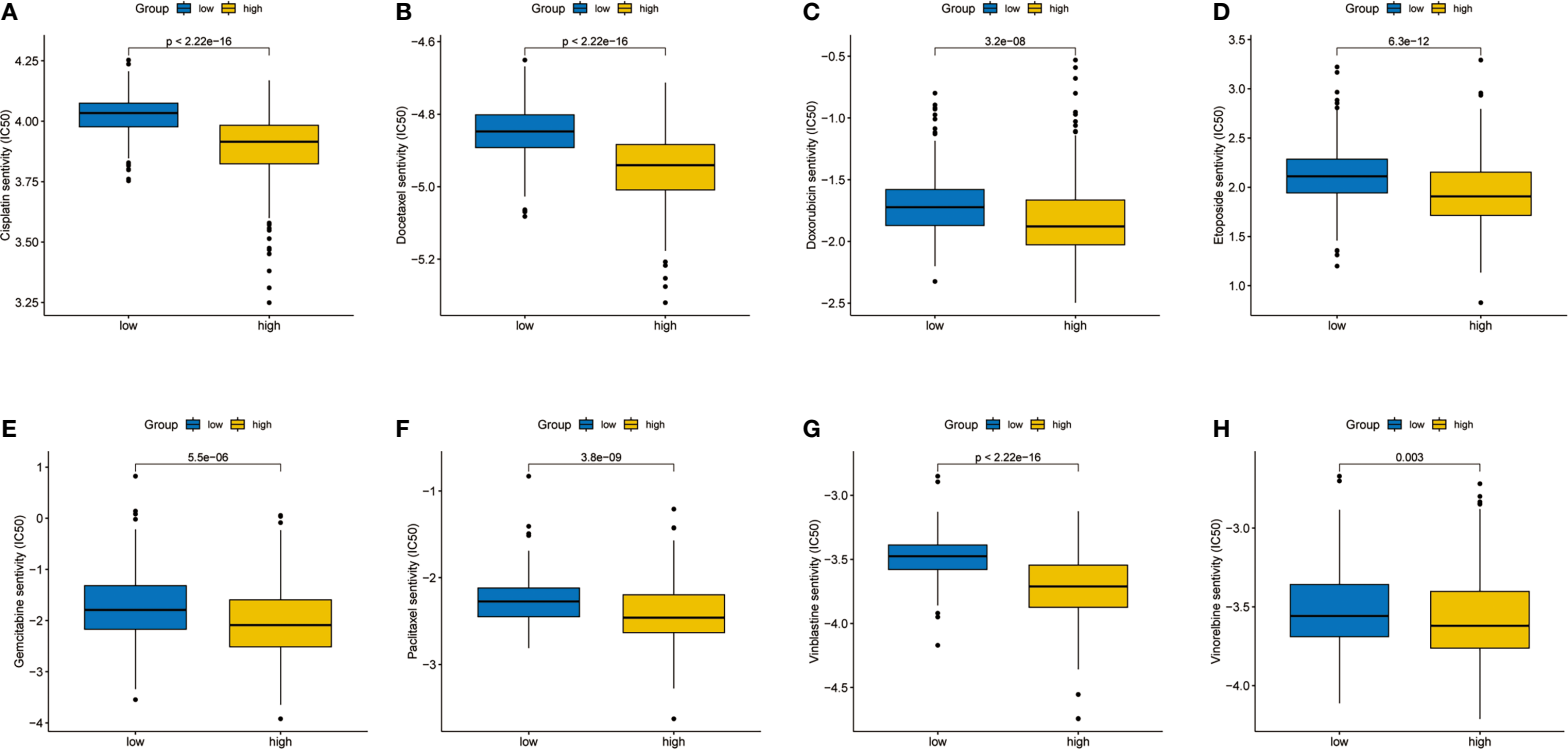
Differences in sensitivity analysis of chemotherapy. **(A)** Cisplatin. **(B)** Docetaxel. **(C)** Doxorubicin. **(D)** Etoposide. **(E)** Gemcitabine. **(F)** Paclitaxel. **(G)** Vinblastine. **(H)** Vinorelbine.

As an important adjuvant treatment, chemotherapy is still indispensable in the treatment of LUAD. We explored the sensitivity of different subgroups to commonly used chemotherapy drugs. The results showed that patients in the high-risk group were more sensitive to many kinds of chemotherapy drugs (**Figures 7A–H**).

### 3.8 ADA knockdown inhibited cell proliferation, migration*, and invasion of lung adenocarcinoma in H1299 and PC9 cell lines*

**Figure 8:**
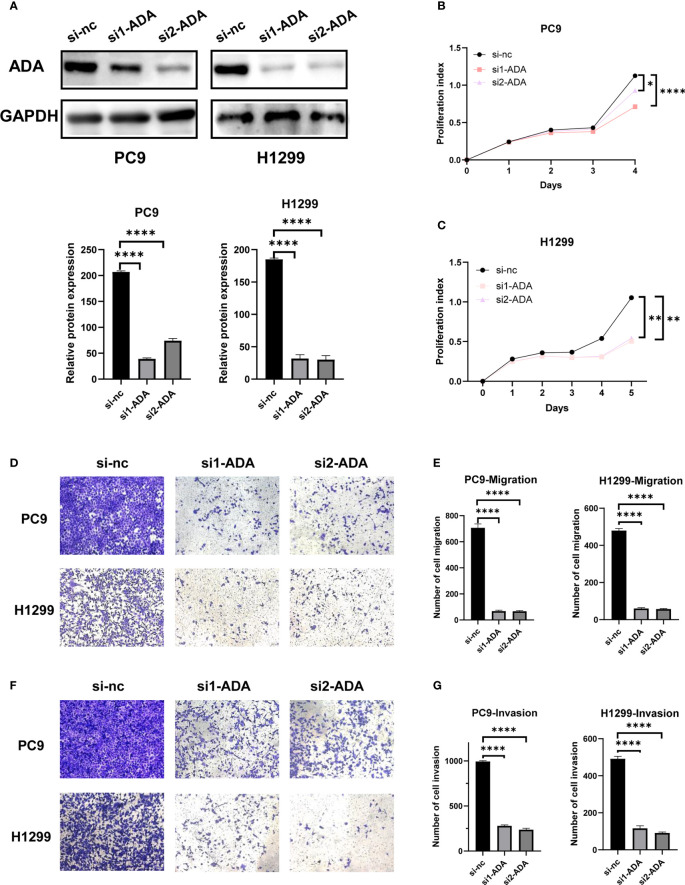
Experimental validation of the role of *ADA* in LUAD cell lines. **(A)** The knocking down efficiency by specific siRNA against *ADA* was confirmed by western blot. **(B, C)** The proliferation ability of PC9 and H1299 cells was measured by the CCK-8 assay after transfecting *ADA* siRNAs. PC9 and H1299 cell migration assay **(D, E)** and invasion assay **(F, G)** were performed in control and siADA groups. *p < 0.05; **p < 0.01; ****p < 0.0001. nc: negative control.

Western blot confirmed that *ADA* was successfully knocked down (**Figure 8A**). The proliferation of H1299 and PC9 cells was significantly inhibited when *ADA* was knocked down in CCK-8 assays (**Figures 8B, C**). Additionally, the knockdown of *ADA* dramatically inhibited migration of lung adenocarcinoma (**Figures 8D, E**) and also inhibited its invasive ability (**Figures 8F, G**). Overall, our findings indicated that *ADA* may promote the proliferation, migration, and invasion of lung adenocarcinoma.

## 4 Discussion

More and more studies have confirmed the important role of the tumor microenvironment in the process of anti-tumor immunity (27). Reactivating the immune system to kill tumor has become the focus of lung cancer treatment research. The emergence of ICIs and adoptive cellular immunotherapy had greatly improved the prognosis of lung cancer patients (28). However, it is frustrating that only a small number of patients respond to immunotherapy (29). How to improve the response of patients to immunotherapy is pressing. T cell is the key factor that destroys tumors during immunotherapy, and its infiltration degree reflects the type of tumor- hot or cold (30), while the effect of immunotherapy is poor for cold tumor due to lack of T cell infiltration (31). Increasing the degree of T cell infiltration and improving the activity of T cells contribute to improving the efficacy of immunotherapy.

In this study, we analyzed the characteristics of T cell proliferation regulators in LUAD, including differences in expression level, prognosis, and tumor microenvironment. The prognostic model was constructed through four key T cell proliferation regulators, which had the excellent ability to predict the survival of LUAD patients and had been verified in the GSE72094. GSEA showed that there were significant differences in pathways between the two different subgroups. There were a large number of cell proliferation and cancer-related pathways in the high-risk group, which meant that the tumor cells in the high-risk group proliferate actively, hence the prognosis was worse. In the low-risk group, a large number of immune pathways were activated, and rich infiltration of immune cells is beneficial to improve the prognosis of patients. Finally, we further explored the role of *ADA* in the progression of LUAD through cytological experiments.

The signature was composed of four T cell proliferation regulators, including *ADA*, *CD19*, *CDK1*, and *CYP27A1*. Adenosine is an important factor in regulating immune response, and its metabolites can interfere with the proliferation and function of T cells. *ADA* can control the immune response by regulating the metabolism of adenosine (32). We found that *ADA* could affect the proliferation, invasion, and migration of LUAD through the knockdown of *ADA*. Therefore, targeting *ADA* may be a new target for the treatment of LUAD. *CD19* is mainly expressed by B cells. It participates in the signal transduction pathway of B cells and mediates the killing of target cells by T cells. Previous experiments have proved that CD19-specific CTL modified by genetic engineering can effectively kill CD19^+^tumor lines (33). *CDK1* is necessary for cell cycle, which promotes the initiation of M phase in mitosis. *CDK1* in tumors is dysfunctional due to genetic changes. Clinical trials targeting *CDK1*, the key enzyme in the process of tumor proliferation, to induce cell cycle arrest are being carried out extensively (34). *CYP27A1* is the key enzyme for the synthesis of 27-hydroxycholesterol. Macrophages treated with 27- hydroxycholesterol significantly inhibit the expansion of T cells and reduce the activity of their immune response (35). In addition, inhibiting the expression of *CYP27A1* can reduce the metastasis of LUAD (36).

Our research also emphasized the key role of TME. The complex components in the TME affect the occurrence, development, and immune response of tumor. Regulating the components in the TME and modifying the immune function of the body is gradually becoming a new breakthrough in the process of tumor treatment (37). We found that the low-risk group had higher abundance of immune cells and richer immune-related pathways, which means that the low-risk group was in a state of immune activation and was conducive to improving the prognosis of LUAD. In addition, the infiltration level of immune cells reflects the efficacy of immunotherapy to a certain extent. According to the TIDE algorithm, compared with the high-risk group, the low-risk group had a better response to immunotherapy, which provides a reliable basis for the clinical application of the TPR signature. Chemotherapy, as a traditional treatment strategy, still plays an important role in cancer. Therefore, we analyzed the sensitivity of LUAD patients to multiple chemotherapy drugs. Surprisingly, the high-risk group patients were more sensitive to many chemotherapeutic drugs. Several phase III clinical trials have confirmed that immunotherapy combined with chemotherapy had brought superior efficacy to NSCLC patients (38–41). In this research, patients in high-risk group were relatively insensitive to immunotherapy, but more sensitive to chemotherapy. Based on this, we speculate that if patients in the high-risk group take the treatment plan of immunotherapy combined with chemotherapy, it may significantly improve their prognosis. In a short, the above results provide accurate treatment suggestions for different patients, and our signature can accurately identify patients who are sensitive to different treatment schemes, thus promoting personalized treatment and reducing the burden of patients.

This study has some limitations. The transcriptome data used in this study were all from public databases. It is necessary to conduct transcriptome sequencing for LUAD patients in the real world to further analyze and verify. In addition, the role of *ADA* in the development of LUAD has only been preliminarily verified by some *in vitro* experiments. Its role *in vivo* is not clear, but it is worth further exploration.

## Conclusion

We have constructed a reliable prognostic signature related to T cell proliferation regulators, which can accurately predict the response of patients to different treatment strategies. *ADA* knockdown experiments showed that *ADA* can be a new target for LUAD treatment.

## Data availability statement

The datasets presented in this study can be found in online repositories. The names of the repository/repositories and accession number(s) can be found in the article/**Supplementary Material**.

## Author contributions

WC, HL, YC: Conceptualization, data curation, formal analysis, writing–original draft, review, and editing. HH: Formal analysis, experimental support, writing–review and editing. WO, S-YW: Conceptualization, supervision, funding acquisition, writing–original draft, project administration, writing–review and editing. All authors contributed to the article and approved the submitted version. 

## Supplementary material

Supplementary Table 1 Differentially expressed genes between Cluster1 and Cluster2.

Supplementary Figure 1 The expression of T cell proliferation regulators in two LUAD clusters.

Supplementary Figure 2 GO and KEGG enrichment. The results of upregulated DEGs in GO **(A)** and KEGG **(B)** enrichment. The results of downregulated DEGs in GO **(C)** and KEGG **(D)** enrichment.

Supplementary Figure 3 Univariate cox regression analysis of T cell proliferation regulators.

Supplementary Figure 4 KM survival analysis and ROC analysis of TPR signature and previous studies for predicting survival of LUAD.

Supplementary Figure 5 Metabolism-related pathways of T cells at the single-cell level by scMetabolism.

## References

[1] Siegel RL Miller KD Fuchs HE Jemal A. Cancer statistics, 2021. CA Cancer J Clin (2021) 71(1):7–33. doi: 10.3322/caac.21654 33433946

[2] Tsao AS Scagliotti GV Bunn PA Jr. Carbone DP Warren GW Bai C. Scientific advances in lung cancer 2015. J Thorac Oncol (2016) 11(5):613–38. doi: 10.1016/j.jtho.2016.03.012 27013409

[3] Miller M Hanna N. Advances in systemic therapy for non-small cell lung cancer. BMJ (2021) 375:n2363. doi: 10.1136/bmj.n2363 34753715

[4] Lim ZF Ma PC. Emerging insights of tumor heterogeneity and drug resistance mechanisms in lung cancer targeted therapy. J Hematol Oncol (2019) 12(1):134. doi: 10.1186/s13045-019-0818-2 31815659 PMC6902404

[5] Passaro A Brahmer J Antonia S Mok T Peters S. Managing resistance to immune checkpoint inhibitors in lung cancer: Treatment and novel strategies. J Clin Oncol (2022) 40(6):598–610. doi: 10.1200/JCO.21.01845 34985992

[6] Kumar BV Connors TJ Farber DL. Human T cell development, localization, and function throughout life. Immunity (2018) 48(2):202–13. doi: 10.1016/j.immuni.2018.01.007 PMC5826622 29466753

[7] Basu A Ramamoorthi G Albert G Gallen C Beyer A Snyder C. Differentiation and regulation of T(H) cells: A balancing act for cancer immunotherapy. Front Immunol (2021) 12:669474. doi: 10.3389/fimmu.2021.669474 34012451 PMC8126720

[8] Farhood B Najafi M Mortezaee K. CD8(+) cytotoxic T lymphocytes in cancer immunotherapy: A review. J Cell Physiol (2019) 234(6):8509–21. doi: 10.1002/jcp.27782 30520029

[9] Tanaka A Sakaguchi S. Regulatory T cells in cancer immunotherapy. Cell Res (2017) 27(1):109–18. doi: 10.1038/cr.2016.151 PMC5223231 27995907

[10] Chen DS Mellman I. Elements of cancer immunity and the cancer-immune set point. Nature (2017) 541(7637):321–30. doi: 10.1038/nature21349 28102259

[11] Liu YT Sun ZJ. Turning cold tumors into hot tumors by improving T-cell infiltration. Theranostics (2021) 11(11):5365–86. doi: 10.7150/thno.58390 PMC8039952 33859752

[12] Legut M Gajic Z Guarino M Daniloski Z Rahman JA Xue X. A genome-scale screen for synthetic drivers of T cell proliferation. Nature (2022) 603(7902):728–35. doi: 10.1038/s41586-022-04494-7 PMC9908437 35296855

[13] Wilkerson MD Hayes DN. ConsensusClusterPlus: a class discovery tool with confidence assessments and item tracking. Bioinformatics (2010) 26(12):1572–3. doi: 10.1093/bioinformatics/btq170 PMC2881355 20427518

[14] Ritchie ME Phipson B Wu D Hu Y Law CW Shi W. Limma powers differential expression analyses for RNA-sequencing and microarray studies. Nucleic Acids Res (2015) 43(7):e47. doi: 10.1093/nar/gkv007 25605792 PMC4402510

[15] Yu G Wang LG Han Y He QY. clusterProfiler: an r package for comparing biological themes among gene clusters. OMICS (2012) 16(5):284–7. doi: 10.1089/omi.2011.0118 PMC3339379 22455463

[16] Dai Z Liu T Liu G Deng Z Yu P Wang B. Identification of clinical and tumor microenvironment characteristics of hypoxia-related risk signature in lung adenocarcinoma. Front Mol Biosci (2021) 8:757421. doi: 10.3389/fmolb.2021.757421 34869590 PMC8634728

[17] Jiang A Chen X Zheng H Liu N Ding Q Li Y. Lipid metabolism-related gene prognostic index (LMRGPI) reveals distinct prognosis and treatment patterns for patients with early-stage pulmonary adenocarcinoma. Int J Med Sci (2022) 19(4):711–28. doi: 10.7150/ijms.71267 PMC9108406 35582412

[18] Li F Song QZ Zhang YF Wang XR Cao LM Li N. Identifying the EMT-related signature to stratify prognosis and evaluate the tumor microenvironment in lung adenocarcinoma. Front Genet (2022) 13:1008416. doi: 10.3389/fgene.2022.1008416 36186418 PMC9523218

[19] Zhao F Wang Z Li Z Liu S Li S. Identifying a lactic acid metabolism-related gene signature contributes to predicting prognosis, immunotherapy efficacy, and tumor microenvironment of lung adenocarcinoma. Front Immunol (2022) 13:980508. doi: 10.3389/fimmu.2022.980508 36275729 PMC9585198

[20] Subramanian A Tamayo P Mootha VK Mukherjee S Ebert BL Gillette MA. Gene set enrichment analysis: a knowledge-based approach for interpreting genome-wide expression profiles. Proc Natl Acad Sci USA (2005) 102(43):15545–50. doi: 10.1073/pnas.0506580102 PMC1239896 16199517

[21] Hanzelmann S Castelo R Guinney J. GSVA: Gene set variation analysis for microarray and RNA-seq data. BMC Bioinf (2013) 14:7. doi: 10.1186/1471-2105-14-7 PMC3618321 23323831

[22] Yoshihara K Shahmoradgoli M Martinez E Vegesna R Kim H Torres-Garcia W. Inferring tumour purity and stromal and immune cell admixture from expression data. Nat Commun (2013) 4:2612. doi: 10.1038/ncomms3612 24113773 PMC3826632

[23] Sun D Wang J Han Y Dong X Ge J Zheng R. TISCH: a comprehensive web resource enabling interactive single-cell transcriptome visualization of tumor microenvironment. Nucleic Acids Res (2021) 49(D1):D1420–30. doi: 10.1093/nar/gkaa1020 PMC7778907 33179754

[24] Wu Y Yang S Ma J Chen Z Song G Rao D. Spatiotemporal immune landscape of colorectal cancer liver metastasis at single-cell level. Cancer Discov (2022) 12(1):134–53. doi: 10.1158/2159-8290.CD-21-0316 34417225

[25] Jiang P Gu S Pan D Fu J Sahu A Hu X. Signatures of T cell dysfunction and exclusion predict cancer immunotherapy response. Nat Med (2018) 24(10):1550–8. doi: 10.1038/s41591-018-0136-1 PMC6487502 30127393

[26] Geeleher P Cox N Huang RS. pRRophetic: an r package for prediction of clinical chemotherapeutic response from tumor gene expression levels. PloS One (2014) 9(9):e107468. doi: 10.1371/journal.pone.0107468 25229481 PMC4167990

[27] Forde PM Kelly RJ Brahmer JR. New strategies in lung cancer: Translating immunotherapy into clinical practice. Clin Cancer Res (2014) 20(5):1067–73. doi: 10.1158/1078-0432.CCR-13-0731 24470514

[28] Herbst RS Morgensztern D Boshoff C. The biology and management of non-small cell lung cancer. Nature (2018) 553(7689):446–54. doi: 10.1038/nature25183 29364287

[29] Darvin P Toor SM Sasidharan Nair V Elkord E. Immune checkpoint inhibitors: Recent progress and potential biomarkers. Exp Mol Med (2018) 50(12):1–11. doi: 10.1038/s12276-018-0191-1 PMC6292890 30546008

[30] Galon J Costes A Sanchez-Cabo F Kirilovsky A Mlecnik B Lagorce-Pages C. Type, density, and location of immune cells within human colorectal tumors predict clinical outcome. Science (2006) 313(5795):1960–4. doi: 10.1126/science.1129139 17008531

[31] Galon J Bruni D. Approaches to treat immune hot, altered and cold tumours with combination immunotherapies. Nat Rev Drug Discov (2019) 18(3):197–218. doi: 10.1038/s41573-018-0007-y 30610226

[32] Bagheri S Saboury AA Haertle T. Adenosine deaminase inhibition. Int J Biol Macromol (2019) 141:1246–57. doi: 10.1016/j.ijbiomac.2019.09.078 31520704

[33] Cooper LJ Topp MS Serrano LM Gonzalez S Chang WC Naranjo A. T-Cell clones can be rendered specific for CD19: Toward the selective augmentation of the graft-versus-B-lineage leukemia effect. Blood (2003) 101(4):1637–44. doi: 10.1182/blood-2002-07-1989 12393484

[34] Asghar U Witkiewicz AK Turner NC Knudsen ES. The history and future of targeting cyclin-dependent kinases in cancer therapy. Nat Rev Drug Discov (2015) 14(2):130–46. doi: 10.1038/nrd4504 PMC4480421 25633797

[35] Ma L Wang L Nelson AT Han C He S Henn MA. 27-hydroxycholesterol acts on myeloid immune cells to induce T cell dysfunction, promoting breast cancer progression. Cancer Lett (2020) 493:266–83. doi: 10.1016/j.canlet.2020.08.020 PMC7572761 32861706

[36] Li X Chen H Zhang L Chen L Wei W Gao S. 27-hydroxycholesterol linked high cholesterol diet to lung adenocarcinoma metastasis. Oncogene (2022) 41(19):2685–95. doi: 10.1038/s41388-022-02285-y PMC9076535 35379924

[37] Wood SL Pernemalm M Crosbie PA Whetton AD. The role of the tumor-microenvironment in lung cancer-metastasis and its relationship to potential therapeutic targets. Cancer Treat Rev (2014) 40(4):558–66. doi: 10.1016/j.ctrv.2013.10.001 24176790

[38] Gandhi L Rodriguez-Abreu D Gadgeel S Esteban E Felip E De Angelis F. Pembrolizumab plus chemotherapy in metastatic non-Small-Cell lung cancer. N Engl J Med (2018) 378(22):2078–92. doi: 10.1056/NEJMoa1801005 29658856

[39] Zhou C Wang Z Sun Y Cao L Ma Z Wu R. Sugemalimab versus placebo, in combination with platinum-based chemotherapy, as first-line treatment of metastatic non-small-cell lung cancer (GEMSTONE-302): Interim and final analyses of a double-blind, randomised, phase 3 clinical trial. Lancet Oncol (2022) 23(2):220–33. doi: 10.1016/S1470-2045(21)00650-1 35038432

[40] Zhou C Chen G Huang Y Zhou J Lin L Feng J. Camrelizumab plus carboplatin and pemetrexed versus chemotherapy alone in chemotherapy-naive patients with advanced non-squamous non-small-cell lung cancer (CameL): A randomised, open-label, multicentre, phase 3 trial. Lancet Respir Med (2021) 9(3):305–14. doi: 10.1016/S2213-2600(20)30365-9 33347829

[41] Nishio M Barlesi F West H Ball S Bordoni R Cobo M. Atezolizumab plus chemotherapy for first-line treatment of nonsquamous NSCLC: Results from the randomized phase 3 IMpower132 trial. J Thorac Oncol (2021) 16(4):653–64. doi: 10.1016/j.jtho.2020.11.025 33333328

