# Supplementary figures and images for: Construction and validation of a T cell proliferation regulator-related signature for predicting prognosis and immunotherapy response in lung adenocarcinoma
Source: Front Immunol. 2023 Apr 4;14:1171145. doi: 10.3389/fimmu.2023.1171145 (PMC10110836; doi:10.3389/fimmu.2023.1171145)

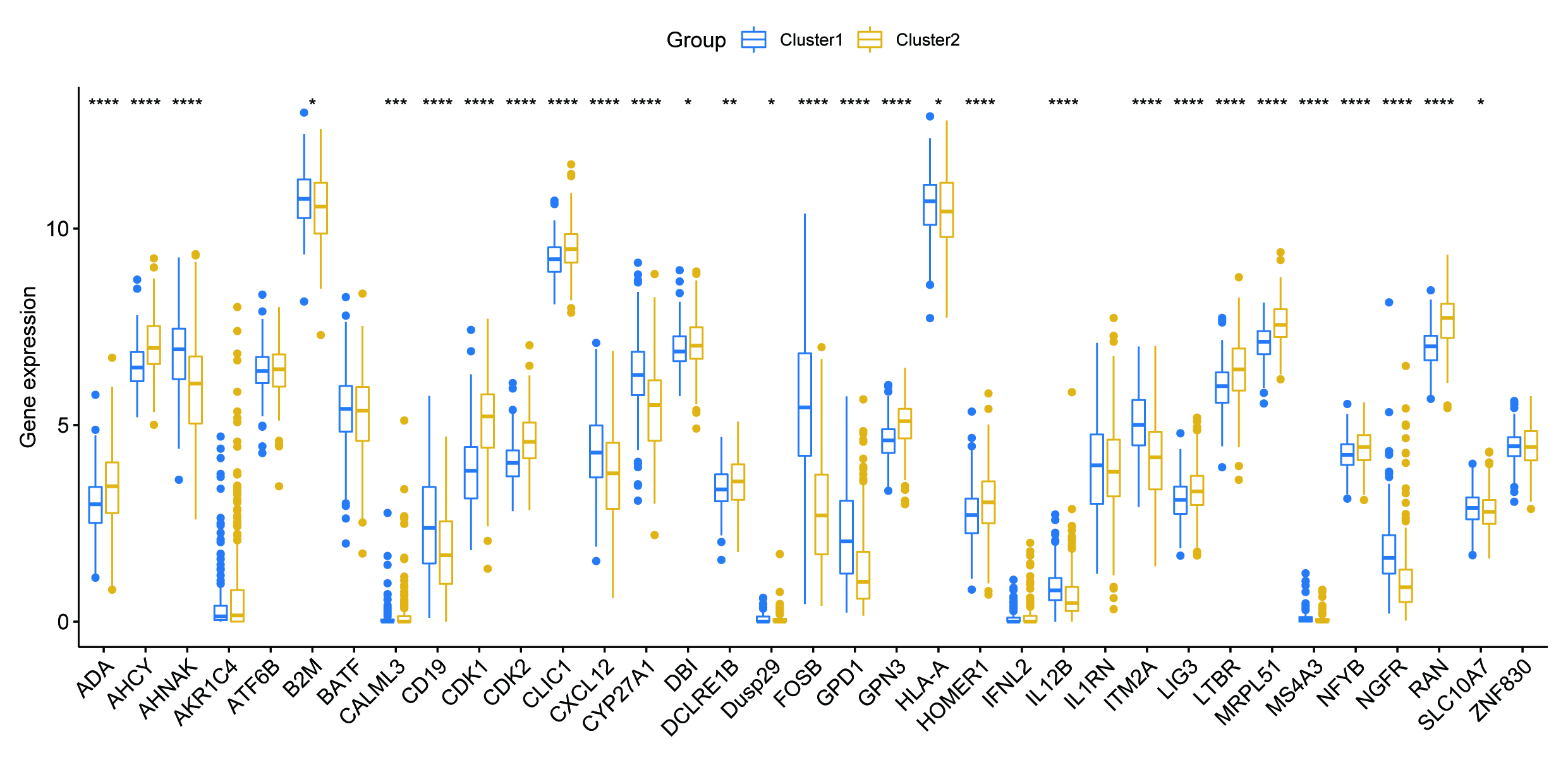

Supplement: Supplementary Figure 1 — The expression of T cell proliferation regulators in two LUAD clusters. [file Image_1.tif]

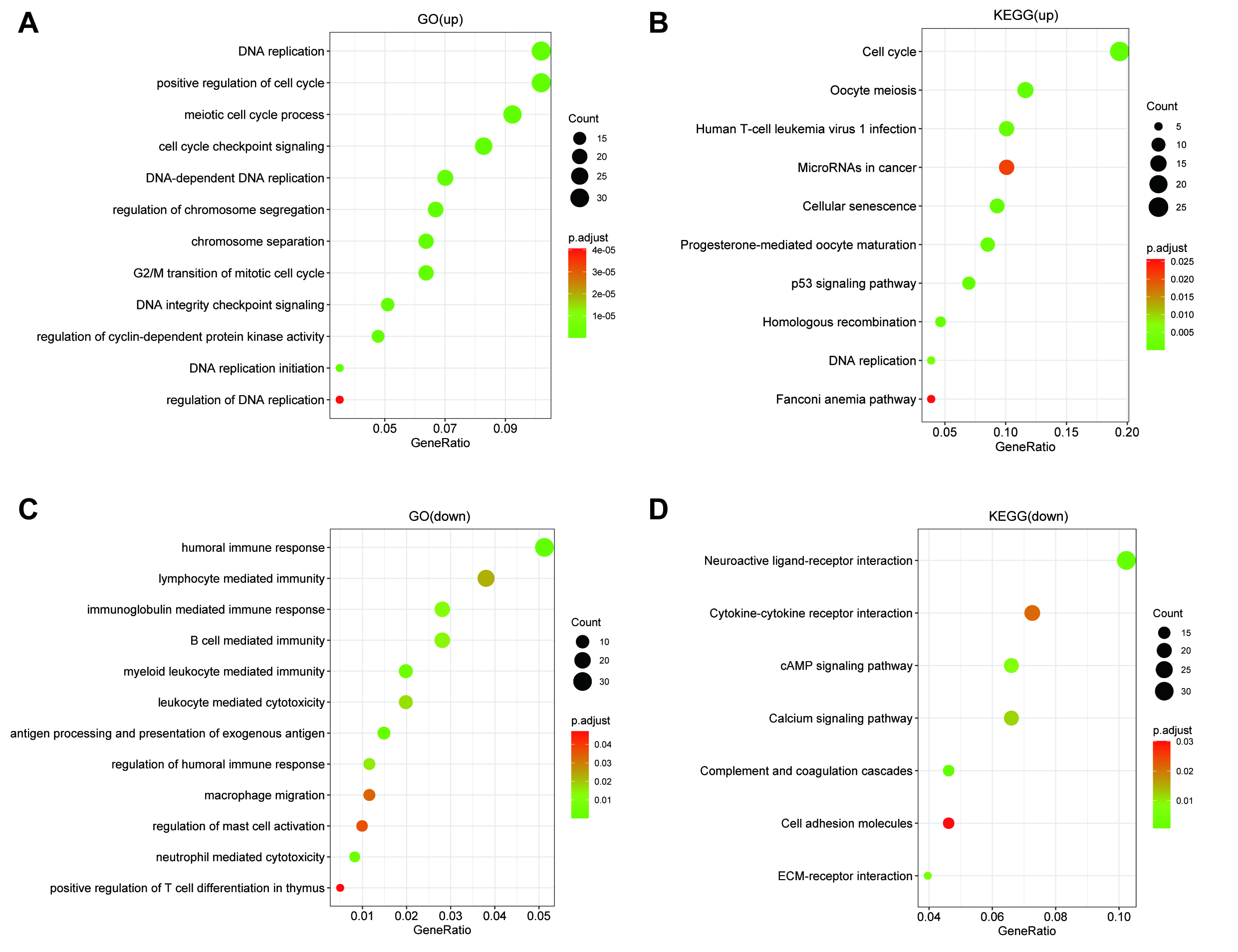

Supplement: Supplementary Figure 2 — GO and KEGG enrichment. The results of upregulated DEGs in GO (A) and KEGG (B) enrichment. The results of downregulated DEGs in GO (C) and KEGG (D) enrichment. [file Image_2.tif]

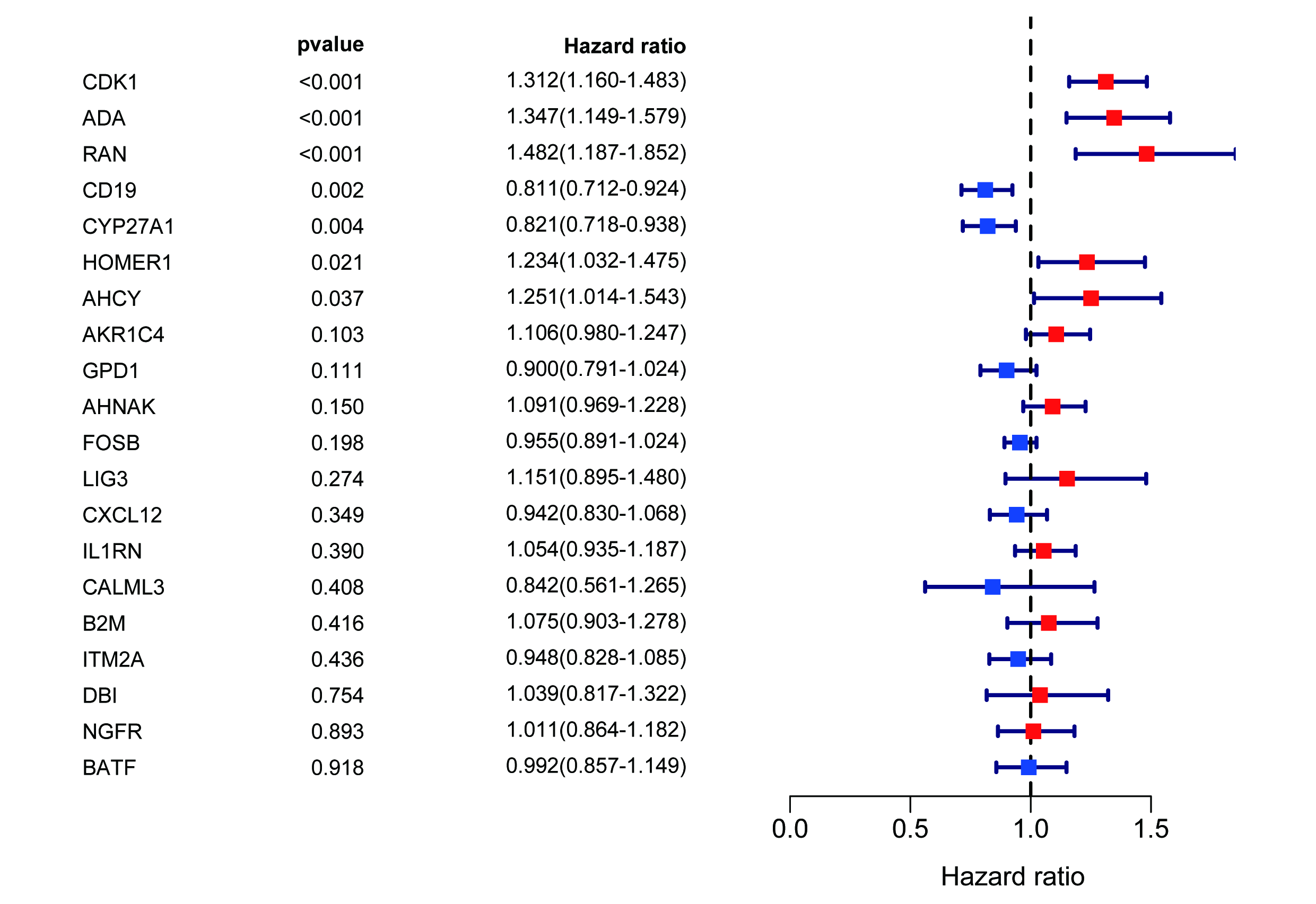

Supplement: Supplementary Figure 3 — Univariate cox regression analysis of T cell proliferation regulators. [file Image_3.tif]

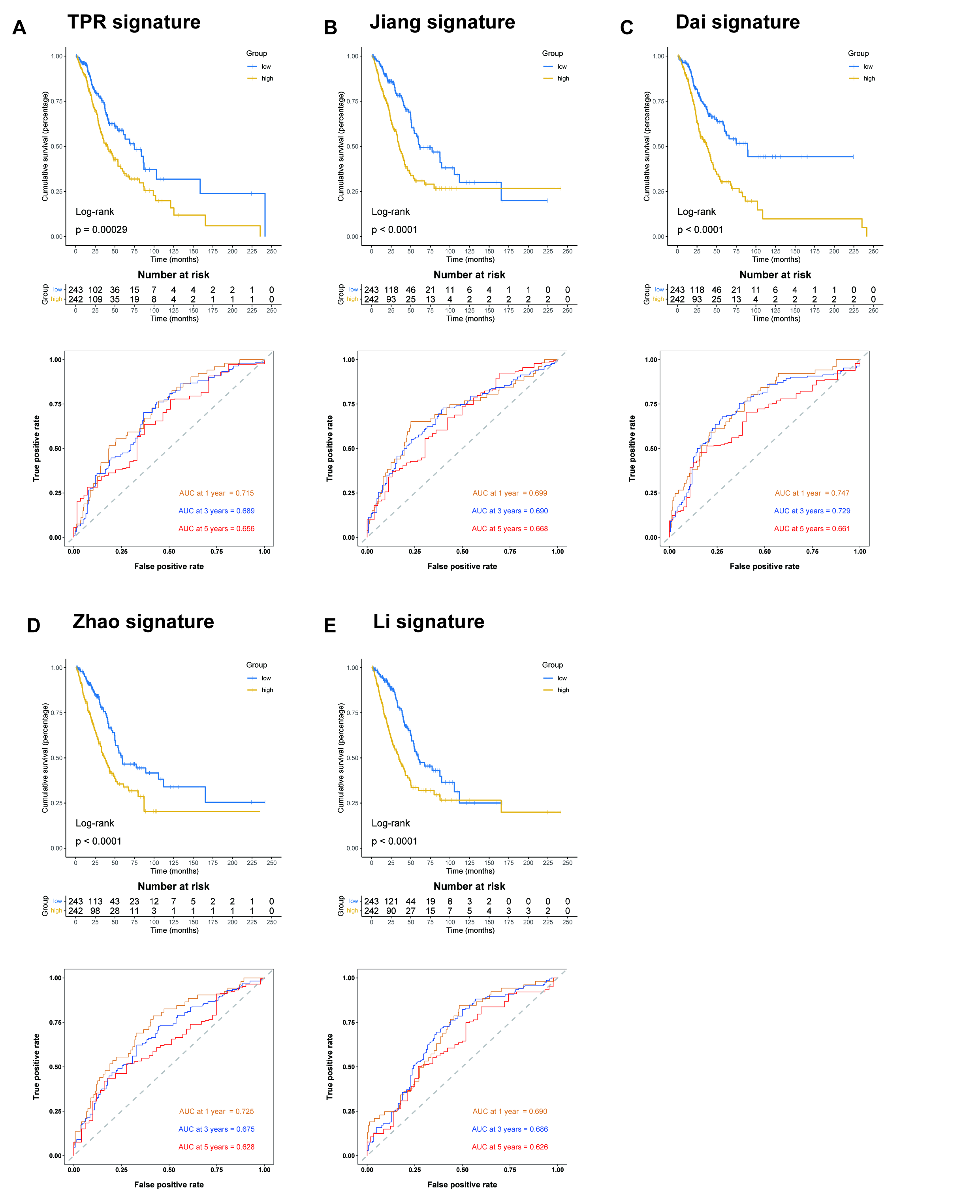

Supplement: Supplementary Figure 4 — KM survival analysis and ROC analysis of TPR signature and previous studies for predicting survival of LUAD. [file Image_4.tif]

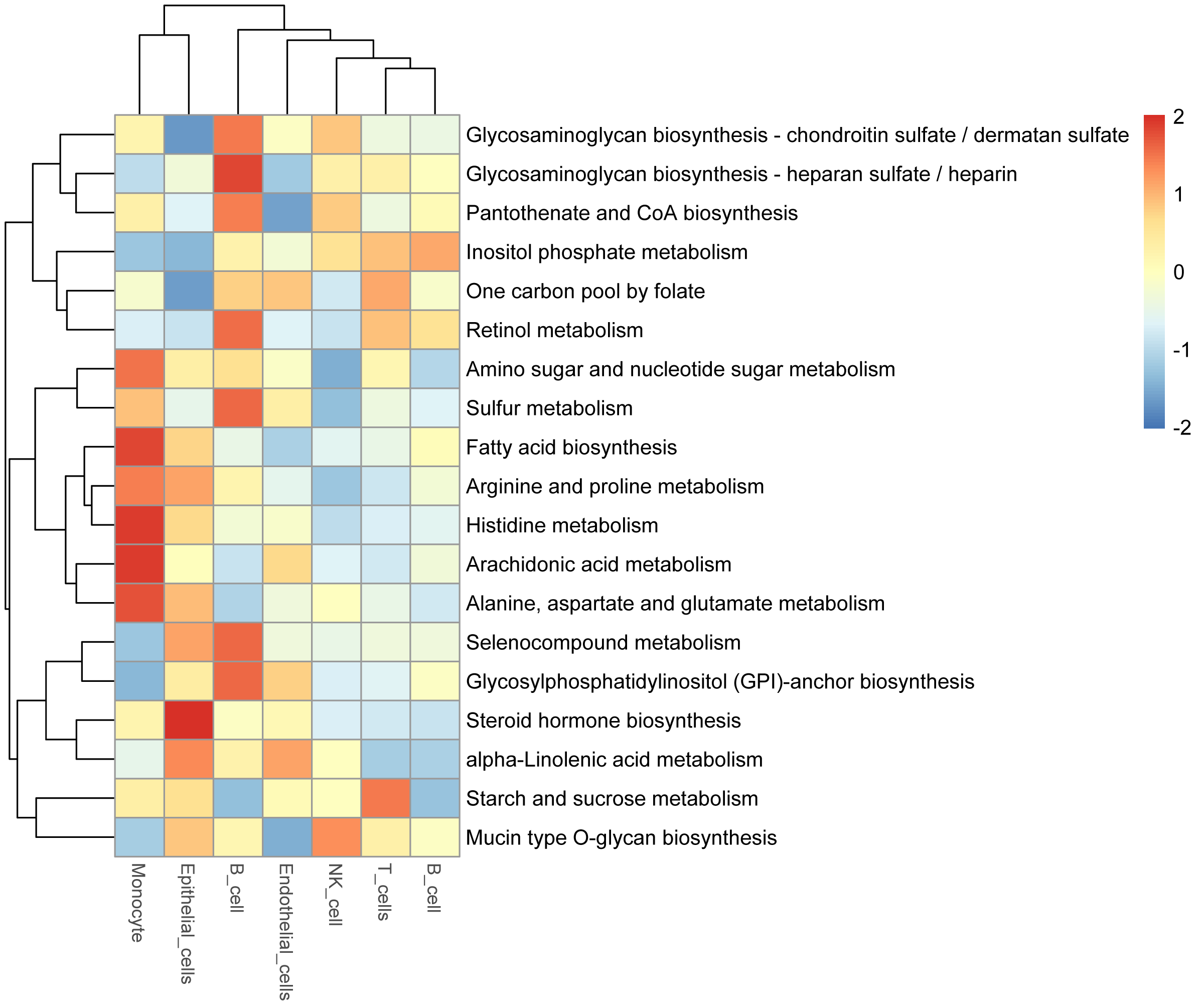

Supplement: Supplementary Figure 5 — Metabolism-related pathways of T cells at the single-cell level by scMetabolism. [file Image_5.tif]
